# Commentary: Peripheral Circulating Exosome-Mediated Delivery of miR-155 as a Novel Mechanism for Acute Lung Inflammation

**DOI:** 10.3389/fmed.2020.00507

**Published:** 2020-09-03

**Authors:** Lin Zhang, Heng Meng, Qing Geng

**Affiliations:** Department of Thoracic Surgery, Renmin Hospital of Wuhan University, Wuhan, China

**Keywords:** exosomes, acute lung injury, macrophages, intercellular communication, miRNAs

Acute lung injury (ALI) is a devastating respiratory disorder characterized by clinically significant hypoxemia, diffuse bilateral pulmonary infiltration, pulmonary edema, decrease in pulmonary compliance, and decrease in functional residual capacity (1). Macrophages, the cells that play a central role in the inflammatory microenvironment of ALI, have been shown to contribute significantly to the inflammatory process of this disease (1). Although the interactions between macrophages and other cells in the inflammatory responses of lung have been well-described previously, the function of exosomes as a mediator of signal transmission in ALI has not been fully elucidated. In a previous issue of *Molecular Therapy*, Jiang et al. described the serum exosomes derived from ALI mice serum that delivered miR-155 to naive mice or naive macrophages to induce inflammation (2). The communicational role of exosomes to carry proinflammatory signals and mediate inflammation was demonstrated by using the adoptively transfer method that involves transferring of immunoreactive molecules from donor to recipient, as was applied in previous research (3).

Exosomes are one kind of extracellular vesicles (EVs) with a diameter of 30–100 nm. Although the regulatory role of microRNAs (miRNAs) in ALI has been corroborated in plenty of studies, their tendency of being degraded in body fluids has been an obstacle to their long-distance delivery. Luckily, exosomes could provide a shelter for these degradable molecules and increase their biological stability. With pleiotropic biological functions, exosomes are natural carriers of protein and nucleic acids, including messenger RNAs (mRNAs) as well as miRNAs. They could be released by various types of cells and are significant mediators in intercellular communications. These vesicles have also been reported to play important roles in pulmonary diseases, including ALI and acute respiratory distress syndrome (4).

Previous research has demonstrated that exosomal miRNAs could promote M1 macrophage polarization and trigger pro-inflammatory effects in ALI (4). Previous studies have also reported that miR-155 could promote lipopolysaccharide (LPS)-induced ALI through the down-regulation of SOCS-1 (5). The findings of Jiang et al. have added to the understanding of the roles of exosomes and exosomal miRNAs in ALI. In their study, exosomal miR-155 played an important regulatory role in the pathogenesis of ALI. Through a series of experiments, including the co-incubation of macrophages with exosomes and a dual luciferase reporter gene assay, they discovered that exosomal miR-155 could mediate cell proliferation *via* targeting SHIP1. Furthermore, their research revealed that exosomal miR-155 could mediate inflammation through suppressing the expression of SOCS1. The proinflammatory effect of the exosomes was also confirmed by *in vivo* experiments in mice. The conclusion was convincing. However, the origin of these regulatory exosomes and their exact recipient cells had not been further clarified.

In humans, ALI could be developed when an individual is exposed to infectious pathogens or to non-infectious noxious stimuli. Thus, it could be divided into an infectious type and a sterile type according to the etiology (3). While non-infectious or sterile ALI is modeled experimentally by hypoxia, acid inhalation, and ventilator-induced baro-trauma, infection-induced ALI could be induced by bacteria, viruses, or various components of these agents, such as LPS or lipoteichoic acid (3). In sterile ALI, the major derivation of exosomes is the epithelial cells. These epithelial cell-derived exosomes could promote macrophage recruitment and lung inflammatory responses *via* their containing miRNAs (3). In contrast, in LPS or gram-negative bacteria-induced infectious ALI, exosomes are mainly secreted by macrophages (6). A further study by Lee et al. showed that, after a bacterial infection, macrophage-derived EVs were dominant in amount (7). Although epithelial cell-derived EVs constituted only a small proportion of the total EVs, they contained rich miRNAs; they could be delivered to alveolar macrophages (AMs) to play important regulatory roles and promote innate immune responses in bacterial lung infections (7). Since exosomes are important mediators of intracellular communication, addressing the question on what the origins of these exosomes are will help to further understand their regulatory mechanisms in ALI. The origin of exosomes could be determined by detection of their surface proteins because exosomes possess the same surface markers as their parent cells do. This approach has been taken in plenty of similar research such as that by Ye et al. (8).

In addition, macrophages are heterogeneous immune cells. There are various subtypes of macrophages in the lungs, with different distributions. The macrophages with the widest distribution are AMs. They are generally located on the luminal surface of the alveolar space and express MerTK, CD64, CD68, CD206, CD11c, MARCO, SiglecF, and F4/80 on the membrane surface. AMs could be easily separated from the bronchoalveolar lavage fluid and constitute the first line of the innate immune system. They are important bactericidal and antigen-presenting cells secreting IL-6 to regulate the immune response (9). In comparison, interstitial macrophages (IMs) are less populous and reside mainly in lung parenchyma or in the vicinity of the bronchi. They have different surface markers, such as MerTK, CD64, CD11b, CD86, CX3CR1, and CD169 and secret IL-1, IL-6, TNF-α, and IL-10, in their steady state (9). IMs could respond to LPS and thus might also be activated in the research by Jiang et al. They could possibly be one of the origins of EVs in the peripheral blood. Previous research has reported that the counts of IMs had been significantly increased in the injured lungs and that IMs were the major subsets of cells in the lung of mice with LPS-induced ALI (10). Thus, it is also interesting to know whether it is IMs or AMs that are the exact target cells of these peripheral blood exosomes.

In summary, the study by Jiang et al. provided further evidence for the concept that exosomes are mediators of intercellular signal transduction, with their cargo miRNAs targeting various pathways (2). The demonstration that ALI mice serum-derived exosomes could target pulmonary macrophages is consistent with the fact that macrophages play a central role in the pathogenesis of ALI. While the origin of these exosomes and the exact subtype of their recipient cells remain unknown, which is partly acknowledged in the study, there is little doubt that circulating serum exosomes play an important part in the induction of pulmonary inflammation during ALI. Overall, the authors have provided enough experimental evidence to support their conclusions.

## Author Contributions

LZ and HM conceived and designed this manuscript. QG was responsible for the revision. All authors contributed to the article and approved the submitted version.

## Conflict of Interest

The authors declare that the research was conducted in the absence of any commercial or financial relationships that could be construed as a potential conflict of interest.

## References

[1] Huang X Xiu H Zhang S Zhang G. The role of macrophages in the pathogenesis of ALI/ARDS. Mediat Inflamm. (2018) 2018:1–8. 10.1155/2018/1264913 29950923 PMC5989173

[2] Jiang K Yang J Guo S Zhao G Wu H Deng G. Peripheral circulating exosome-mediated delivery of miR-155 as a novel mechanism for acute lung inflammation. Mol Ther. (2019) 27:1758–71. 10.1016/j.ymthe.2019.07.003 31405809 PMC6822235

[3] Lee H Zhang D Laskin DL Jin Y. Functional evidence of pulmonary extracellular vesicles in infectious and noninfectious lung inflammation. J Immunol. (2018) 201:1500–9. 10.4049/jimmunol.1800264 29997122 PMC6109965

[4] Lanyu Z Feilong H. Emerging role of extracellular vesicles in lung injury and inflammation. Biomed Pharmacother. (2019) 113:108748. 10.1016/j.biopha.2019.108748 30877881

[5] Wang W Liu Z Su J Chen WS Wang XW Bai SX. Macrophage micro-RNA-155 promotes lipopolysaccharide-induced acute lung injury in mice and rats. Am J Physiol Lung Cell Mol Physiol. (2016) 311:L494–506. 10.1152/ajplung.00001.2016 27371731

[6] Soni S Wilson MR O'Dea KP Yoshida M Katbeh U Woods SJ. Alveolar macrophage-derived microvesicles mediate acute lung injury. Thorax. (2016) 71:1020–9. 10.1136/thoraxjnl-2015-208032 27287089 PMC5099194

[7] Lee H Groot M Pinilla-Vera M Fredenburgh LE Jin Y. Identification of miRNA-rich vesicles in bronchoalveolar lavage fluid: insights into the function and heterogeneity of extracellular vesicles. J Control Release. (2019) 294:43–52. 10.1016/j.jconrel.2018.12.008 30529727 PMC6372374

[8] Ye C Li H Bao M Zhuo R Jiang G Wang W. Alveolar macrophage - derived exosomes modulate severity and outcome of acute lung injury. Aging. (2020) 12:6120–8. 10.18632/aging.103010 32259794 PMC7185135

[9] Mukaida N Nosaka T Nakamoto Y Baba T. Lung macrophages: multifunctional regulator cells for metastatic cells. Int J Mol Sci. (2019) 20:116. 10.3390/ijms20010116 30597969 PMC6337639

[10] Chen J Wang S Fu R Zhou M Zhang T Pan W. RIP3 dependent NLRP3 inflammasome activation is implicated in acute lung injury in mice. J Transl Med. (2018) 16:233. 10.1186/s12967-018-1606-4 30126430 PMC6102827

